# [Bmim]Br Accelerated One-Pot Three-Component Cascade Protocol for the Construction of Spirooxindole–Pyrrolidine Heterocyclic Hybrids

**DOI:** 10.3390/molecules25204779

**Published:** 2020-10-18

**Authors:** Raju Suresh Kumar, Dhaifallah M. Al-thamili, Abdulrahman I. Almansour, Natarajan Arumugam, Necmi Dege

**Affiliations:** 1 Department of Chemistry, College of Science, King Saud University, P.O. Box 2455, Riyadh 11451, Saudi Arabia; daife54321@hotmail.com (D.M.A.-t.); almansor@ksu.edu.sa (A.I.A.); anatarajan@ksu.edu.sa (N.A.); 2 Department of Physics, Faculty of Arts and Sciences, Ondokuz Mayıs University, Samsun 55139, Turkey; necmid@omu.edu.tr

**Keywords:** one-pot cascade reactions, 1,3-dipolar cycloaddition, ionic liquid, selectivity, spirooxindole–pyrrolidines

## Abstract

Our synthetic approach for the assembly of structurally complex spirooxindole heterocyclic hybrids was based on an ionic liquid, [bmim]Br mediated one-pot three-component cascade reaction strategy involving 1,3-dipolar cycloaddition reaction of *N*-1-(2-pyridinylmethyl)-3,5-bis[(*E*)-arylmethylidene]tetrahydro-4(1*H*)-pyridinones and azomethine ylide generated in situ from isatin and L-phenyl alanine, affording a series of spirooxindole–pyrrolidine heterocyclic hybrids in good-to-excellent yields. In addition to serving as the reaction medium, [bmim]Br also functioned as a catalyst in this cycloaddition reaction and hence accelerated the reaction rate affording the cycloadducts in short reaction time.

## 1. Introduction

A swift construction of molecular complexity from easily available and simple starting precursors advances the field of chemistry. One-pot multicomponent cascade reactions are highly effective and prevailing protocols that incorporate multiple bond-forming events, thus constituting an attractive division of organic chemistry and attaining great interest, as these reactions permit the construction of molecules with noteworthy structural complexity from simple and easily available starting materials [1,2,3,4]. These one-pot cascade reactions have advantages such as atom economy, operational simplicity, and high reaction efficiency, in addition affording better overall yields when compared with the classical single-step transformations [5]. These reactions avoid the isolation and purification of intermediates, consequently leading to reduction in pollution and, as a result, come under the category of green chemistry [6]. Specifically, these reactions are useful for the construction of vastly functionalized and valuable building blocks comprising several stereogenic centers, and also for the synthesis of biologically intriguing natural products and heterocyclic compounds.

1,3-dipolar cycloaddition is a powerful synthetic approach for the formation of a variety of heterocycles; this eco-compatible synthetic procedure has recently received a great deal of attention and considerable progress has been achieved in this area [7,8,9]. 1,3-dipolar cycloaddition reaction of azomethine ylides with activated electron deficient alkenes offers a proficient methodology for synthesizing pyrrolidine heterocyclic hybrids in a regio- and stereoselective fashion [10,11,12].

Ionic liquids play a most important role in advancing synthetic chemistry and in recent past years have gained immense attention as green solvents, catalysts, and reagents due to their identifications and tunable characteristics. Moreover, their high thermal and chemical stability, negligible vapor pressure, good solvating ability, non-coordinating nature, and ease of recyclability make them a good choice for diverse chemical reactions. It is noteworthy to mention that ionic liquids act both as solvents and catalysts in diverse transformations [13,14,15].

The major advantage of spiroheterocycles is their inherent three-dimensional nature and associated ability to project functionality in all three dimensions. Noteworthy interactions of a ligand with a three-dimensional binding site can be achieved more easily with a spirocyclic core than with planar aromatic systems [16]. Due to their wide occurrence in many natural products, highly functionalized spirooxindole pyrrolidines have gained much importance and are the central skeleton for several alkaloids and pharmacologically important compounds [17,18]. In recent years, we have been involved in the synthesis of spiroheterocyclic hybrids derived through one-pot cascade reaction [19,20,21,22,23]. Herein, we commence exploring the synthesis of structurally complex highly substituted pyrrolidines through ionic liquid mediated cascade reaction and report the results in this article.

## 2. Results and Discussion

**Scheme 1:**
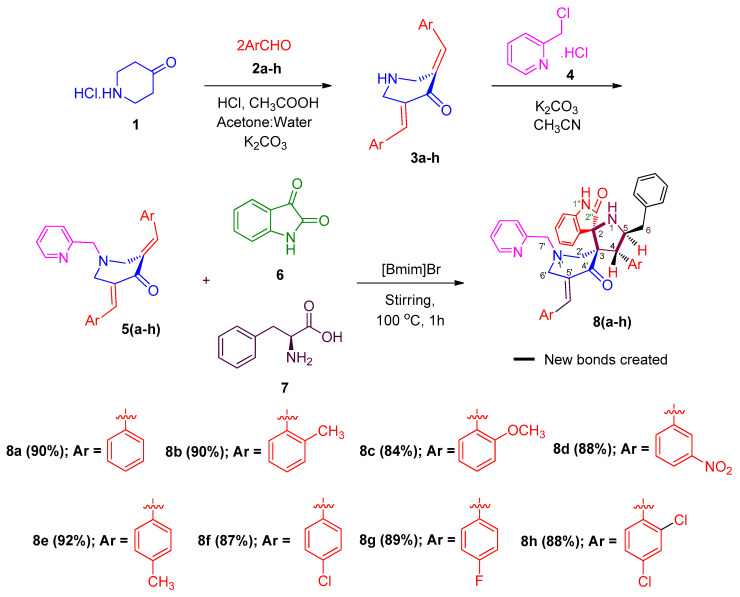
Synthesis of spirooxindole–pyrrolidine heterocyclic hybrids **8a**–**h**.

The methodology for the synthesis of spirooxindole heterocyclic hybrids is based on a one-pot three-component cascade reaction strategy employing *N*-1-(2-pyridinylmethyl)-3,5-bis[(*E*)-arylmethylidene]tetrahydro-4(1*H*)-pyridinones **5a**–**h**, isatin **6,** and L-proline **7** involving a key 1,3-dipolar cycloaddition reaction, as outlined in Scheme 1. The starting precursors **5a**–**h** were prepared as reported by us earlier [24] through the alkylation of compounds **3a**–**h** with an equivalent amount of 2-(chloromethyl)pyridine hydrochloride in the presence of K_2_CO_3,_ as shown in Scheme 1. To begin with the one-pot three-component cycloaddition reaction, primarily in order to optimize the reaction conditions, a characteristic reaction was performed involving **5e**, isatin **6,** and l-phenylalanine **7** with different solvents viz. ethanol, methanol, dioxane, methanol:dioxane (1:1 *v*/*v*), and acetonitrile (Table 1). The reaction of isatin **6** and L-phenylalanine **7** via decarboxylative condensation afforded the azomethine ylide which, in turn, added regioselectivity to **5e**. In a representative reaction, an equimolar mixture of **5e**, **6**, and **7** was dissolved in 5 mL of ethanol and heated under reflux with constant stirring for 6 h. When all the starting substrates had been consumed completely, as obvious from thin-layer chromatography (TLC) analysis, the reaction mixture was poured into ice cold water (50 mL). The solid, thus precipitated, was filtered and washed with water to obtain **8e** in 67% yield. The reactions in methanol and dioxane afforded moderate and good yield (45 and 85%) of **8e**, while the product was obtained only in traces in acetonitrile. Among these solvents, the reaction in methanol afforded a better yield of the product **8e** (85%) when compared to other solvents. As the prime objective in current organic synthesis is to develop new synthetic methods employing green solvents, we planned to conduct the same reaction in an ionic liquid, [bmim]Br. As expected, an excellent yield of **8e** (92%) has been observed in [bmim]Br in a considerably shorter reaction time (1 h) (Scheme 1, Table 1). Then, all other reactions employing dipolarophiles with different substitutions on the aryl rings were executed with the ionic liquid. Good yields of the products were observed irrespective of the substitutions on the aryl ring of **5**.

**Figure 1:**
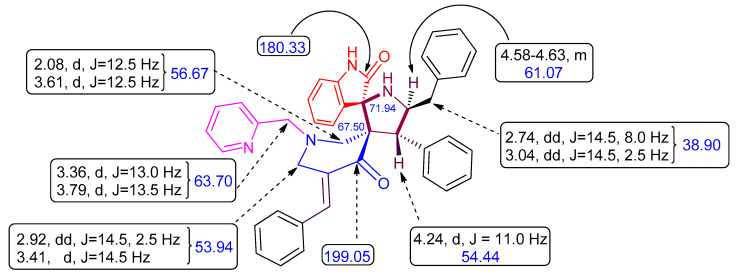
Selected ^1^H and ^13^C-NMR chemical shifts of **8e.**

A careful structural elucidation of the spirooxindole–pyrrolidine heterocyclic hybrids was accomplished with the help of spectroscopic data considering a representative case, **8e** (vide Supplementary Material). In the IR spectrum of **8e**, the key infrared absorption peaks at υ_max_ 2359, 2342, 1713, and 1589 cm^−1^ is referred to N-H, C=O, and C=C groups. In the ^1^H-NMR spectrum, the H-4 proton appears as a doublet at 4.24 ppm (*J* = 11.0 Hz), while the H-5 proton appears as a multiplet at 4.58–4.63 ppm. H-6 protons appear as doublet of doublets at 2.74 ppm (*J* = 14.5, 8.0 Hz) and 3.04 ppm (*J* = 14.5, 2.5 Hz). C,H-COSY correlation of H-4, H-5 and H-6 protons assigns the carbon signals at 54.44, 61.07, and 38.90 ppm to C-4, C-5, and C-6, respectively. The two doublets at 2.08 and 3.61 ppm were due to the piperidone ring protons 2′-CH_2_, whereas the other ring protons (6′-CH_2_) appeared as a doublet of doublets and a doublet at 2.92 ppm (*J* = 14.5, 2.5 Hz) and 3.41 ppm (*J* = 14.5 Hz). The doublets at 3.36 ppm (*J* = 13.0 Hz) and 3.79 ppm (*J* = 13.5Hz) were assigned to 7′-CH_2_. From HMQC spectra, the carbon signals at 56.67, 53.94, and 63.70 ppm were assigned to C-2′, C-6′, and C-7′, respectively. The carbonyl carbons of oxindole and piperidone rings appear at 180.33 ppm and 199.05 ppm. The selected ^1^H and ^13^C chemical shifts of **8e** are shown in Figure 1. The structure of other cycloadducts was also derived by similar considerations.

**Figure 2:**
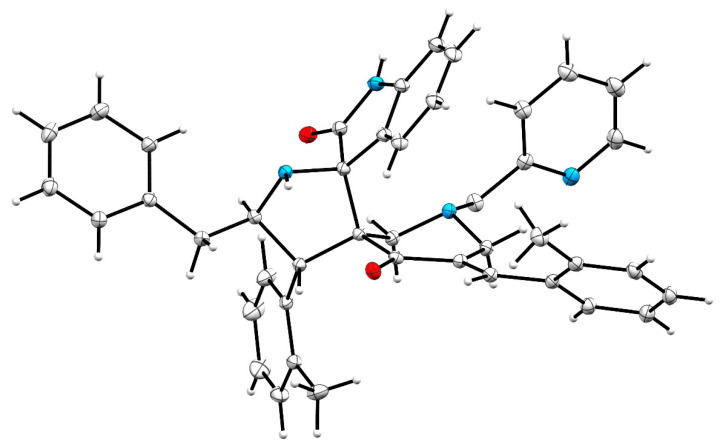
Oak Ridge Thermal Ellipsoid Plot (ORTEL) diagram of **8e.**

The structure of one of the cycloadduct **8b** was further confirmed by the single crystal X-ray analysis (Figure 2) [25].

**Scheme 2:**
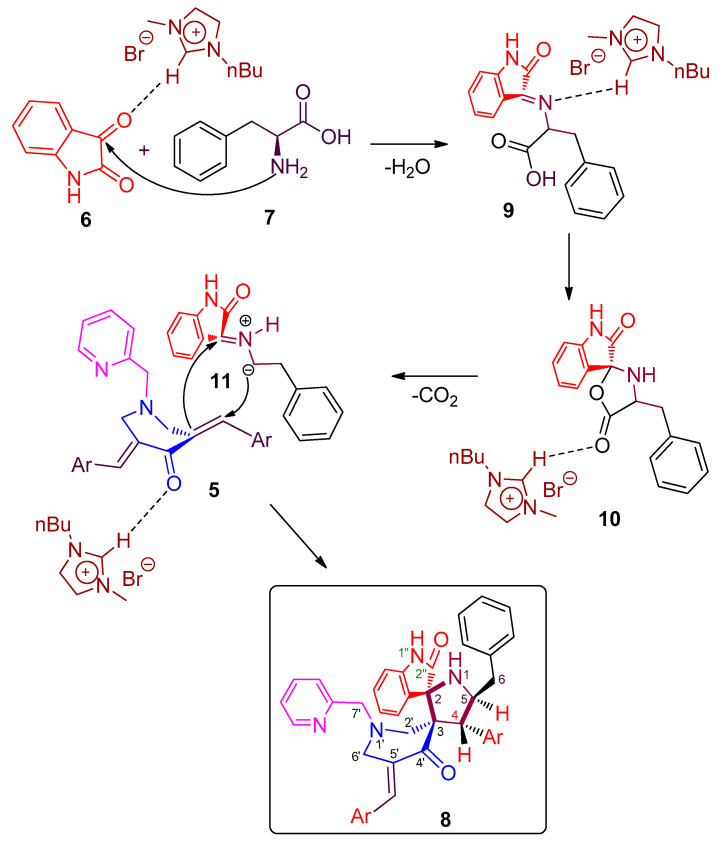
Feasible mechanistic pathway for the formation of spirooxindole–pyrrolidine hybrids **8a**–**h.**

A feasible mechanistic pathway for the assembly of spirooxindole–pyrrolidine hybrids **8a**–**h** is shown in Scheme 2. Firstly, the reaction of isatin **6** and L-phenylalanine **7** affords the imine **9** via the elimination of a molecule of water, and the hydrogen bonding interaction of [bmim]Br with the carbonyl group of isatin facilitates the attack of -NH_2_ of L-phenylalanine. Again, the hydrogen bonding interaction of [bmim]Br with the -C=O group of the cyclic intermediate **10** derived from **9** facilitates the formation of azomethine ylide **11** via decarboxylation. The interaction of [bmim]Br with the carbonyl group **5** perhaps stimulates the exocyclic double bond, allowing the addition of azomethine ylide **11** and resulting in the construction of **8a**–**h**. It is evident from Scheme 2 that the association of ionic liquid with both the starting substrates and intermediates could be responsible for the rate acceleration furnishing the heterocyclic hybrids **8a**–**h** in excellent yields in a shorter reaction time. This one-pot three-component cascade reaction progressed in a highly regio- and stereocontrolled fashion, affording a sole diastereoisomer of the cycloadduct **8a**–**h**. The spirooxindole–pyrrolidine heterocyclic hybrids were constructed via a route which involved the creation of two C-C and one C-N bonds with four contiguous stereocenters.

## 3. Materials and Methods

The reagents and solvents employed in the existing study were got from commercial dealers. All the reactions were observed by thin-layer chromatography (TLC) on silica gel. Melting points of the synthesized compounds were measured using open capillary tubes and are uncorrected. NMR spectra were recorded on a Jeol 500 MHz instrument (Tokyo, Japan) while the FT-IR spectra were documented on a Perkin Elmer system 2000 FT-IR instrument (KBr) (Shelton, AL, USA). Mass spectra of these compounds were measured on a DART-ToF-MS mass spectrometer (Jeol, MA, USA). Elemental analyses of the synthesized compounds were done on a Perkin Elmer 2400 Series II Elemental CHNS analyzer (Waltham, MA, USA).

### General Procedure for the Synthesis of 4-(Aryl)-5-benzylpyrrolo(spiro [2.3″]oxindole)-spiro[3.3′]-5′-(arylmethylidene)-1′-N-(pyridinylmethyl)piperidin-4′-one 8a–h

An equimolar mixture of *N*-1-(2-pyridinylmethyl)-3,5-bis[(*E*)-arylmethylidene]tetrahydro-4(1*H*)-pyridinone **5**, isatin **6**, and phenylalanine **7** in 100 mg of [bmim]Br was heated with constant stirring at 100 °C. Progress of this cycloaddition reaction was monitored after every 15 min time interval, and TLC analysis of the reaction mixture revealed the complete disappearance of the starting materials at 1 h of reaction time. Ethyl acetate (10 mL) was added to the reaction mixture, followed by stirring for 15 min. The ethyl acetate layer was then washed with water (50 mL) and evaporated under reduced pressure. The residue was dried in vacuo and purified by column chromatography, eluting with a 6:4 petroleum ether–ethyl acetate mixture to afford the product **8** in good yield.

4-Phenyl-5-benzylpyrrolo(spiro[2.3′′]-oxindole)-spiro-[3.3′]-5′-(phenylmethylidene)-1′-*N*-(pyridinylmethyl)piperidin-4′-one (**8a**): Obtained as white solid (90%); mp = 98–100 °C; IR (KBr): 2358, 2341, 1712, 1589 cm^−1^; ^1^H-NMR (500 MHz, CDCl_3_): δ_H_ 2.04 (d, 1H, *J* = 12.5 Hz, 2′-CH_2_), 2.75 (dd, 1H, *J* = 14.5, 8.0 Hz, 6-CH_2_), 2.90 (dd, 1H, *J* = 14.0, 2.5 Hz, 6′-CH_2_), 3.03 (dd, 1H, *J* = 14.0, 3.0 Hz, 6-CH_2_), 3.34 (d, 1H, *J* = 13.0 Hz, 7′-CH_2_), 3.40 (d, 1H, *J* = 14.0 Hz, 6′-CH_2_), 3.61(dd, 1H, *J* = 12.5, 2.0 Hz, 2′-CH_2_), 3.75 (d, 1H, *J* = 14.0 Hz, 7′-CH_2_) 4.26 (d, 1H, *J* = 11.0 Hz, 4-CH), 4.61–4.64 (m,1H, 5-CH), 6.56 (d, 1H, *J* = 7.5 Hz, ArH), 6.86–7.04 (m, 8H, ArH), 7.15–7.25 (m, 10H, ArH), 7.32–7.46 (m, 4H, ArH), 7.88 (s, 1H, NH), 8.40–8.42 (m, 1H, ArH). ^13^C-NMR (125 MHz, CDCl_3_): δ_C_ 39.08, 53.80, 54.78, 56.73, 61.10, 63.58, 67.86, 71.95, 109.06, 122.04, 122.17, 123.26, 126.35, 126.73, 127.04, 128.25, 128.42, 128.56, 128.69, 128.90, 129.23, 129.45, 129.87, 130.01, 133.18, 134.93, 136.28, 137.30, 137.72, 138.46, 141.44, 149.01, 157.45, 180.38, 199.19. Mass: 617 [M^+^]. Anal. calc. for C_41_H_36_N_4_O_2_: C, 79.84; H, 5.88; N, 9.08. Found: C, 79.95; H, 5.72; N, 9.21%.

4-(2-Methylphenyl)-5-benzylpyrrolo(spiro[2.3″]oxindole)spiro[3.3′]-5′-(2-methylphenyl-methylidene)-1′-*N*-(pyridinylmethyl)piperidin-4′-one (**8b**): Obtained as white solid (90%); mp = 108–111 °C; IR (KBr): 2357, 2338, 1710, 1588 cm^−1^; ^1^H-NMR (500 MHz, CDCl_3_): δ_H_ 2.02 (d, 1H, *J* = 12.5 Hz, 2′-CH_2_), 2.09 (s, 3H, CH_3_), 2.27 (s, 3H, CH_3_), 2.78 (dd, 1H, *J* = 13.5, 8.0 Hz, 6-CH_2_), 2.85 (dd, 1H, *J* = 15.0, 2.5 Hz, 6′-CH_2_), 2.95 (dd, 1H, *J* = 14.0, 4.0 Hz, 6-CH_2_), 3.19–3.24 (m, 2H, 7′-CH_2_, 6′-CH_2_), 3.44 (d, 1H, *J* = 12.5 Hz, 2′-CH_2_), 3.60 (d, 1H, *J* = 14.0 Hz, 7′-CH_2_), 4.44 (d, 1H, *J* = 9.5 Hz, 4-CH), 4.76–4.81 (m, 1H, 5-CH), 6.64 (d, 2H, *J* = 8.0 Hz, ArH), 6.78 (d, 1H, *J* = 8.0 Hz, ArH), 6.86–6.93 (m, 2H, ArH), 6.97–7.27 (m, 13H, ArH), 7.37–7.40 (m, 1H, ArH), 7.50 (s, 1H, ArH), 7.82 (d, 1H, *J* = 7.5 Hz, ArH), 7.95 (s, 1H, NH), 8.35–8.37 (m, 1H, ArH). ^13^C-NMR (125 MHz, CDCl_3_): δ_C_ 20.08, 21.30, 39.78, 51.99, 54.55, 58.33, 63.30, 64.22, 65.50, 73.60, 109.24, 122.05, 122.38, 122.64, 125.52, 126.11, 126.23, 126.72, 126.85, 128.23, 128.35, 128.51, 128.75, 129.15, 129.20, 130.15, 130.42, 132.51, 134.02, 136.16, 136.64, 137.36, 137.84, 138.13, 138.92, 141.85, 148.98, 157.69, 178.88, 199.49. Mass: 645 [M^+^]. Anal. calc. for C_43_H_40_N_4_O_2_: C, 80.10; H, 6.25; N, 8.69. Found: C, 80.29; H, 6.33; N, 8.55%.

4-(2-Methoxyphenyl)-5-benzylpyrrolo(spiro[2.3″]oxindole)spiro[3.3′]-5′-(2-methoxy-phenylmethylidene) 1′-*N*-(pyridinylmethyl)piperidin-4′-one (**8c**): Obtained as reddish brown solid (84%); mp = 90–93 °C; IR (KBr): 2358, 2341, 1710, 1587 cm^−1^; ^1^H-NMR (500 MHz, CDCl_3_): δ_H_ 2.06 (d, 1H, *J* = 12.5 Hz, 2′-CH_2_), 2.81 (dd, 1H, *J* = 13.0, 8.0 Hz, 6-CH_2_), 2.87 (dd, 1H, *J* = 14.0, 2.5 Hz, 6′-CH_2_), 2.97 (dd, 1H, *J* = 14.0, 4.0 Hz, 6-CH_2_), 3.35–3.44 (m, 1H, 7′-CH_2_), 3.46 (s, 3H, OCH_3_), 3.49–3.57 (m, 1H, 6′-CH_2_), 3.70 (s, 3H, OCH_3_), 3.74 (d, 1H, *J* = 12.5 Hz, 2′-CH_2_), 3.83 (d, 1H, *J* = 13.5 Hz, 7′-CH_2_), 4.04 (d, 1H, *J* = 9.0 Hz, 4-CH), 4.70–4.80 (m,1H, 5-CH), 6.64 (d, 2H, *J* = 7.5 Hz, ArH), 6.80 (d, 1H, *J* = 8.0 Hz, ArH), 6.89–6.98 (m, 2H, ArH), 7.00–7.43 (m, 14H, ArH), 7.53 (s, 1H, ArH), 7.84 (d, 1H, *J* = 7.0 Hz, ArH), 7.96 (s, 1H, NH), 8.38–8.41 (m, 1H, ArH). ^13^C-NMR (125 MHz, CDCl_3_): δ_C_ 39.81, 52.03, 54.72, 55.41, 55.89, 58.51, 63.43, 64.31, 66.02, 73.64, 109.30, 122.36, 122.71, 122.85, 125.53, 126.07, 126.32, 127.12, 127.56, 128.34, 128.55, 128.91, 128.97, 129.61, 130.25, 130.44, 132.63, 134.12, 136.13, 136.42, 137.43, 140.56, 141.73, 148.99, 157.72, 158.10, 160.95, 178.97, 199.55. Mass: 677 [M^+^]. Anal. calc. for C_43_H_40_N_4_O_4_: C, 76.31; H, 5.96; N, 8.28. Found: C, 76.42; H, 5.80; N, 8.39%.

4-(3-Nitrophenyl)-5-benzylpyrrolo(spiro[2.3″]oxindole)spiro[3.3′]-5′-(3-nitrophenyl methylidene)-1′-*N*-(pyridinylmethyl)piperidin-4′-one (**8d**): Obtained as white solid (88%); mp = 100–103 °C; IR (KBr): 2360, 2341, 1711, 1587 cm^−1^; ^1^H-NMR (500 MHz, CDCl_3_): δ_H_ 1.96 (d, 1H, *J* = 12.0 Hz, 2′-CH_2_), 2.87–2.97 (m, 3H, 6-CH_2_, 6′-CH_2_), 3.29 (d, 1H, *J* = 15.0 Hz, 7′-CH_2_), 3.34 (d, 1H, *J* = 13.0 Hz, 6′-CH_2_), 3.58 (d, 1H, *J* = 13.0 Hz, 2′-CH_2_), 3.73 (d, 1H, *J* = 13.0 Hz, 7′-CH_2_), 4.33 (d, 1H, *J* = 11.0 Hz, 4-CH), 4.66–4.70 (m, 1H, 5-CH), 6.64 (d, 1H, *J* = 7.5 Hz, ArH), 6.89–7.18 (m, 11H, ArH), 7.31–7.68 (m, 6H, ArH), 7.81 (s, 1H, NH), 8.01–8.21 (m, 3H, ArH), 8.43–8.51 (m, 1H, ArH). ^13^C-NMR (125 MHz, CDCl_3_): δ_C_ 39.77, 53.34, 54.40, 56.30, 61.91, 63.20, 67.80, 71.81, 109.47, 122.17, 122.50, 122.66, 123.25, 123.35, 123.60, 123.67, 124.09, 124.54, 126.54, 126.64, 128.47, 129.32, 129.76, 134.21, 134.42, 135.26, 135.37, 135.97, 136.32, 136.48, 136.61, 137.72, 140.02, 141.69, 148.42, 149.29, 156.51, 179.96, 198.52. Mass: 707 [M^+^]. Anal. calc. for C_41_H_34_N_6_O_6_: C, 69.68; H, 4.85; N, 11.89. Found: C, 69.80; H, 4.94; N, 11.72%.

4-(4-Methylphenyl)-5-benzylpyrrolo(spiro[2.3″]oxindole)spiro[3.3′]-5′-(4-methylphenyl-methylidene)-1′-*N*-(pyridinylmethyl)piperidin-4′-one (**8e**): Obtained as white solid (92%); mp = 95–98 °C; IR (KBr): 2359, 2342, 1713, 1589 cm^−1^; ^1^H-NMR (500 MHz, CDCl_3_): δ_H_ 2.08 (d, 1H, *J* = 12.5 Hz, 2′-CH_2_), 2.26 (s, 3H, CH_3_), 2.36 (s, 3H, CH_3_), 2.74 (dd, 1H, *J* = 14.5, 8.0 Hz, 6-CH_2_), 2.92 (dd, 1H, *J* = 14.5, 2.5 Hz, 6′-CH_2_), 3.04 (dd, 1H, *J* = 14.5, 2.5 Hz, 6-CH_2_), 3.36 (d, 1H, *J* = 13.0Hz, 7′-CH_2_), 3.41 (d, 1H, *J* = 14.5 Hz, 6′-CH_2_), 3.61(d, 1H, *J* = 12.5 Hz, 2′-CH_2_), 3.79 (d, 1H, *J* = 13.5Hz, 7′-CH_2_), 4.24 (d, 1H, *J* = 11.0 Hz, 4-CH), 4.58–4.63 (m,1H, 5-CH), 6.55 (d, 1H, *J* = 8.0 Hz, ArH), 6.81 (d, 2H, *J* = 8.0 Hz, ArH), 6.86–7.24 (m, 14H, ArH), 7.32 (d, 2H, *J* = 7.5 Hz, ArH), 7.46–7.49 (m, 1H, ArH), 7.68 (s, 1H, NH), 8.44–8.46 (m, 1H, ArH). ^13^C-NMR (125 MHz, CDCl_3_): δ_C_ 21.11, 21.28, 38.90, 53.94, 54.44, 56.67, 61.07, 63.70, 67.50, 71.94, 108.91, 121.89, 122.03, 123.12, 126.19, 126.58, 128.28, 128.71, 128.95, 129.17, 129.33, 129.60, 129.74, 130.14, 131.97, 132.03, 132.10, 134.49, 136.19, 136.45, 137.33, 138.48, 138.98, 141.30, 148.90, 157.48, 180.33, 199.05. Mass: 660 [M^+^]. Anal. calc. for C_43_H_40_N_4_O_2_: C, 80.10; H, 6.25; N, 8.69. Found: C, 80.23; H, 6.17; N, 8.81%.

4-(4-Chlorophenyl)-5-benzylpyrrolo(spiro[2.3″]oxindole)spiro[3.3′]-5′-(4-chlorophenyl-methylidene)-1′-*N*-(pyridinylmethyl)piperidin-4′-one (**8f**): Obtained as yellow solid (87%); mp = 93–96 °C; IR (KBr): 2359, 2340, 1712, 1588 cm^−1^; ^1^H-NMR (500 MHz, CDCl_3_): δ_H_ 1.99 (d, 1H, *J* = 12.5 Hz, 2′-CH_2_), 2.75 (dd, 1H, *J* = 14.5, 8.0 Hz, 6-CH_2_), 2.88 (dd, 1H, *J* = 14.5, 2.5 Hz, 6′-CH_2_), 2.98 (dd, 1H, *J* = 14.5, 3.5 Hz, 6-CH_2_), 3.32–3.37 (m, 2H, 6′-CH_2_, 7′-CH_2_), 3.57 (dd, 1H, *J* = 13.0, 2.5 Hz, 2′-CH_2_), 3.76 (d, 1H, *J* = 13.0 Hz, 7′-CH_2_), 4.21 (d, 1H, *J* = 11.0 Hz, 4-CH), 4.54–4.59 (m, 1H, 5-CH), 6.55 (d, 1H, *J* = 8.5 Hz, ArH), 6.78–6.89 (m, 5H, ArH), 6.97–7.38 (m, 14H, ArH), 7.48–7.52 (m, 1H, ArH), 7.63 (s, 1H, NH), 8.45–8.46 (m, 1H, ArH). ^13^C-NMR (125 MHz, CDCl_3_): δ_C_ 39.16, 53.67, 53.93, 56.63, 61.30, 63.54, 67.75, 71.81, 109.10, 122.02, 122.34, 123.30, 126.45, 126.69, 128.45, 128.57, 128.72, 128.99, 129.39, 131.19, 132.88, 133.23, 133.46, 136.01, 136.31, 138.10, 138.45, 141.43, 149.24, 157.21, 180.09, 198.85. Mass: 685 [M^+^]. Anal. calc. for C_41_H_34_Cl_2_N_4_O_2_: C, 71.82; H, 5.00; N, 8.17. Found: C, 71.74; H, 5.12; N, 8.26%.

4-(4-Fluorophenyl)-5-benzylpyrrolo(spiro[2.3″]oxindole)spiro[3.3′]-5′-(4-fluorophenyl-methylidene)-1′-*N*-(pyridinylmethyl)piperidin-4′-one (**8g**): Obtained as white solid (89%); mp = 98–101 °C; IR (KBr): 2357, 2339, 1712, 1588 cm^−1^; ^1^H-NMR (500 MHz, CDCl_3_): δ_H_ 2.00 (d, 1H, *J* = 13.0 Hz, 2′-CH_2_), 2.76 (dd, 1H, *J* = 14.0, 7.5 Hz, 6-CH_2_), 2.89 (dd, 1H, *J* = 15.0, 3.0 Hz, 6′-CH_2_), 2.99 (dd, 1H, *J* = 14.0, 3.5 Hz, 6-CH_2_), 3.32–3.36 (m, 2H, 6′-CH_2_, 7′-CH_2_), 3.56 (dd, 1H, *J* = 12.5, 2.0 Hz, 2′-CH_2_), 3.75 (d, 1H, *J* = 13.0 Hz, 7′-CH_2_), 4.22 (d, 1H, *J* = 11.0 Hz, 4-CH), 4.54–4.59 (m, 1H, 5-CH), 6.55 (d, 1H, *J* = 8.0 Hz, ArH), 6.85–6.92 (m, 8H, ArH), 6.97–7.10 (m, 5H, ArH), 7.14–7.22 (m, 4H, ArH), 7.36–7.40 (m, 2H, ArH), 7.45–7.49 (m,1H, ArH), 7.73 (s, 1H, NH), 8.43–8.44 (m, 1H, ArH). ^13^C-NMR (125 MHz, CDCl_3_): δ_C_ 39.20, 53.71, 54.03, 56.64, 61.43, 63.57, 67.62, 71.90, 109.08, 115.30, 115.47, 122.02, 122.28, 123.27, 126.42, 126.68, 128.43, 128.94, 129.10, 129.39, 130.10, 131.91, 131.97, 133.05, 133.60, 136.22, 136.27, 138.27, 141.51, 149.16, 157.28, 160.20, 161.20, 180.33, 199.08. Mass: 653 [M^+^]. Anal. calc. for C_41_H_34_F_2_N_4_O_2_: C, 75.44; H, 5.25; N, 8.58. Found: C, 75.58; H, 5.36; N, 8.49%.

4-(2,4-Dichlorophenyl)-5-benzylpyrrolo(spiro[2.3″]oxindole)spiro[3.3′]-5′-(2,4-dichloro-phenylmethylidene)-1′-*N*-(pyridinylmethyl)piperidin-4′-one (**8h**): Obtained as yellow solid (88%); mp = 103–106 °C; IR (KBr): 2358, 2341, 1711, 1588 cm^−1^; ^1^H-NMR (500 MHz, CDCl_3_): δ_H_ 2.11 (d, 1H, *J* = 12.5 Hz, 2′-CH_2_), 2.87–2.91 (m, 1H, 6-CH_2_), 2.94–3.00 (m, 2H, 6′-CH_2_, 6-CH_2_), 3.09 (d, 1H, *J* = 12.5 Hz, 7′-CH_2_), 3.15 (d, 1H, *J* = 15.0 Hz, 6′-CH_2_), 3.23 (d, 1H, *J* = 13.0 Hz, 2′-CH_2_), 3.54 (d, 1H, *J* = 14.0 Hz, 7′-CH_2_) 4.58 (d, 1H, *J* = 9.5 Hz, 4-CH), 4.68–4.73 (m,1H, 5-CH), 6.63–6.65 (m, 2H, ArH), 6.73 (d, 1H, *J* = 8.5 Hz, ArH), 6.86–7.42 (m, 14H, ArH), 7.67 (s, 1H, NH), 7.82–7.85 (m, 2H, ArH), 8.37–8.38 (m, 1H, ArH). ^13^C-NMR (125 MHz, CDCl_3_): δ_C_ 40.41, 52.79, 54.61, 57.79, 63.62, 64.40, 64.68, 74.50, 109.36, 122.22, 122.40, 122.87, 126.33, 126.62, 126.82, 127.10, 127.41, 128.32, 128.87, 128.95, 129.07, 129.80, 130.48, 131.72, 132.98, 133.69, 133.99, 135.23, 135.93, 136.30, 136.61, 138.21, 141.64, 149.08, 157.39, 177.86, 198.72. Mass: 755 [M^+^]. Anal. calc. for C_41_H_32_Cl_4_N_4_O_2_: C, 65.26; H, 4.27; N, 7.43. Found: C, 65.41; H, 4.38; N, 7.51%.

## 4. Conclusions

In conclusion, we have constructed a series of novel spirooxindole–pyrrolidine heterocyclic hybrids employing an ionic liquid accelerated one-pot three-component cascade reaction protocol involving a key 1,3-dipolar cycloaddition reaction. Azomethine ylide generated in situ from isatin and phenylalanine reacted with a new class of functionalized dipolarophiles affording the cycloadducts. The ionic liquid served both as the reaction medium and catalyst and thus accelerated the reaction rate, affording good yields of the products. The structural elucidation of these compounds was done by NMR spectroscopy and mass spectrometric data and further confirmed by X-ray diffraction analysis. Investigation of anticancer potential of these structurally intriguing spiroheterocycles will be the subject of our future research.

## Figures and Tables

**Table 1:** Solvent optimization for the synthesis of spirooxindole–pyrrolidine heterocyclic hybrid **8e**.

Entry	Solvents	Temp (°C)	Time (h)	Yield ^a^ (%)
1	Ethanol	80	6	67
2	Methanol	70	2	85
3	Dioxane	100	10	45
4	Methanol: Dioxane	100	10	51
5	Acetonitrile	80	10	Traces
6	[bmim]Br	100	1	92

^a^ Isolated yield.

## Supplementary Materials

The following are available online. Figures S1–S7: NMR and FT-IR spectra of a representative compound.
