# Supplementary material for: [Bmim]Br Accelerated One-Pot Three-Component Cascade Protocol for the Construction of Spirooxindole–Pyrrolidine Heterocyclic Hybrids
Source: Molecules. 2020 Oct 18;25(20):4779. doi: 10.3390/molecules25204779 (PMC7587566; doi:10.3390/molecules25204779)
Supplement: Supplementary file 1 [file molecules-25-04779-s001.pdf]

Supplementary data

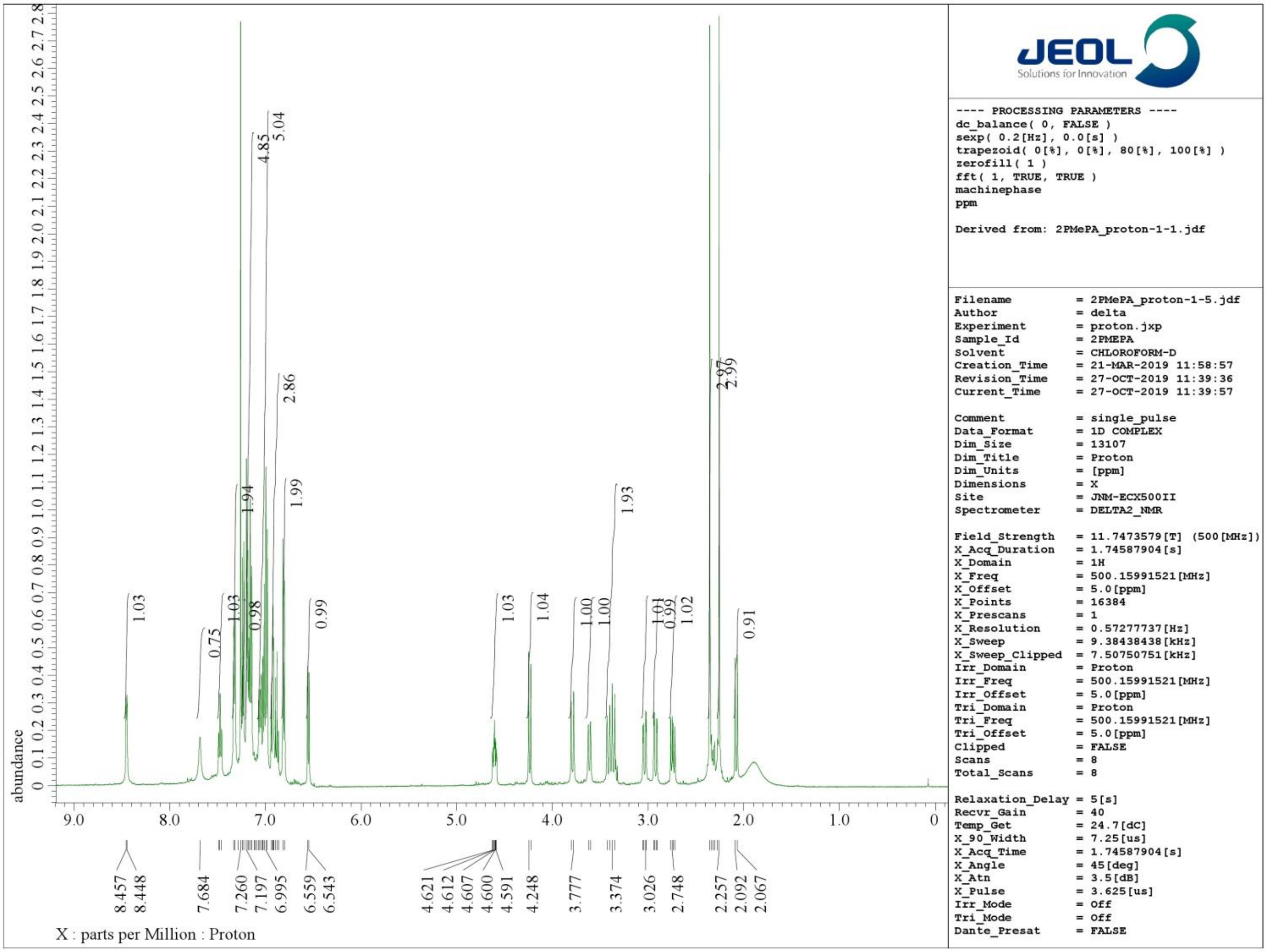

Fig. S1. ^{1}H NMR spectrum of **8e**

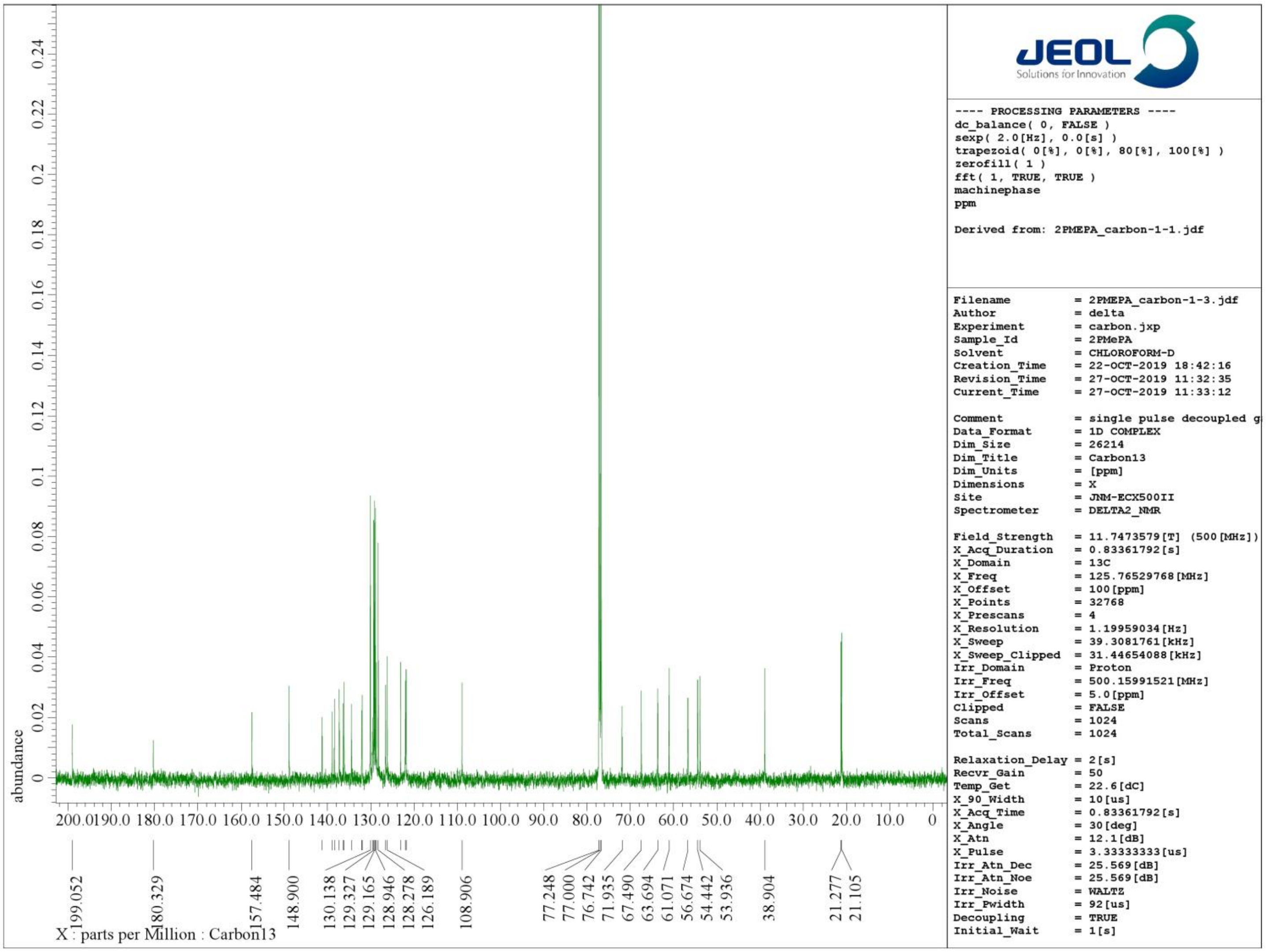

Fig. S2. ^{13}C NMR spectrum of **8e**

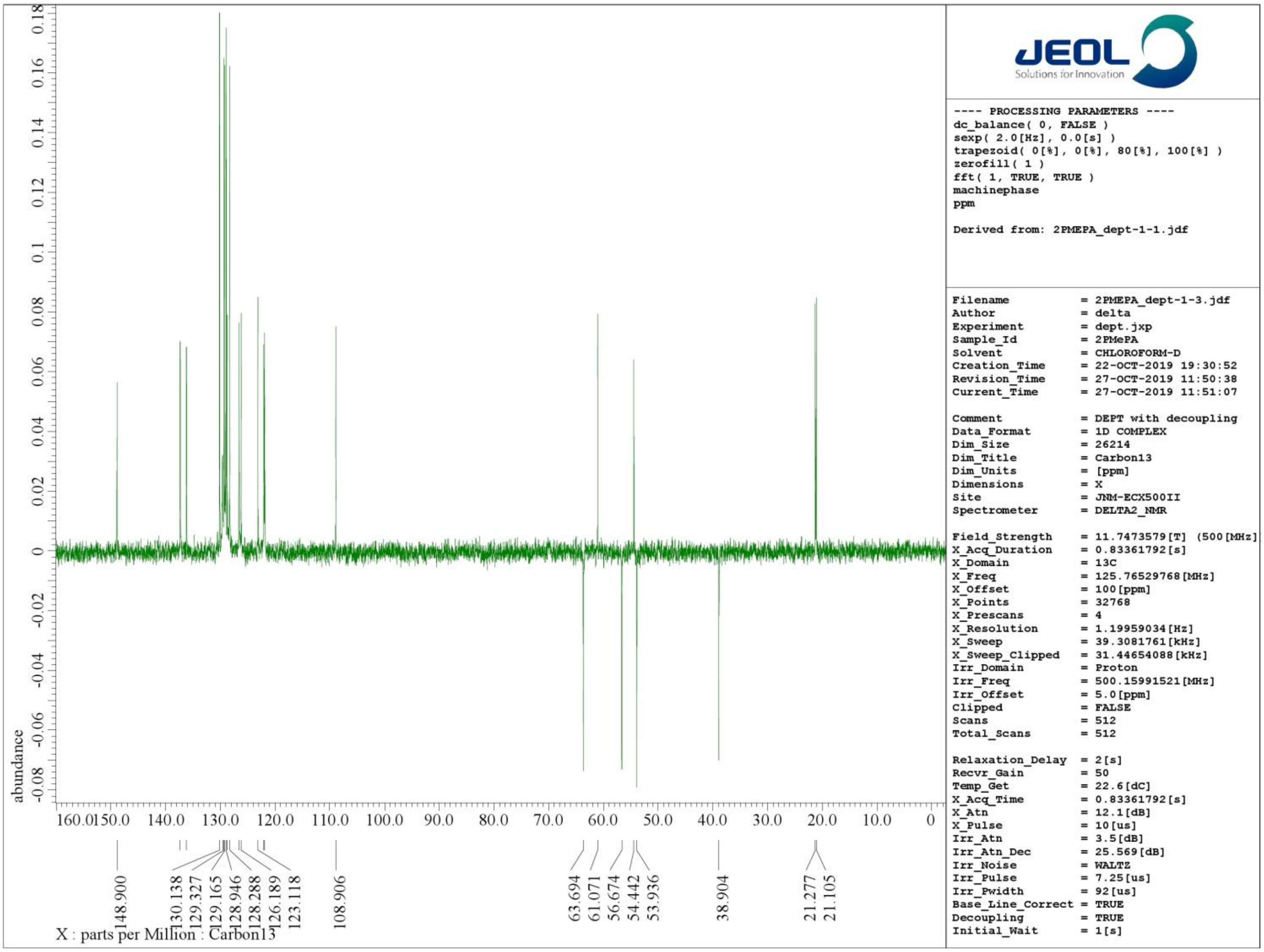

Fig. S3. DEPT 135 NMR spectrum of **8e**

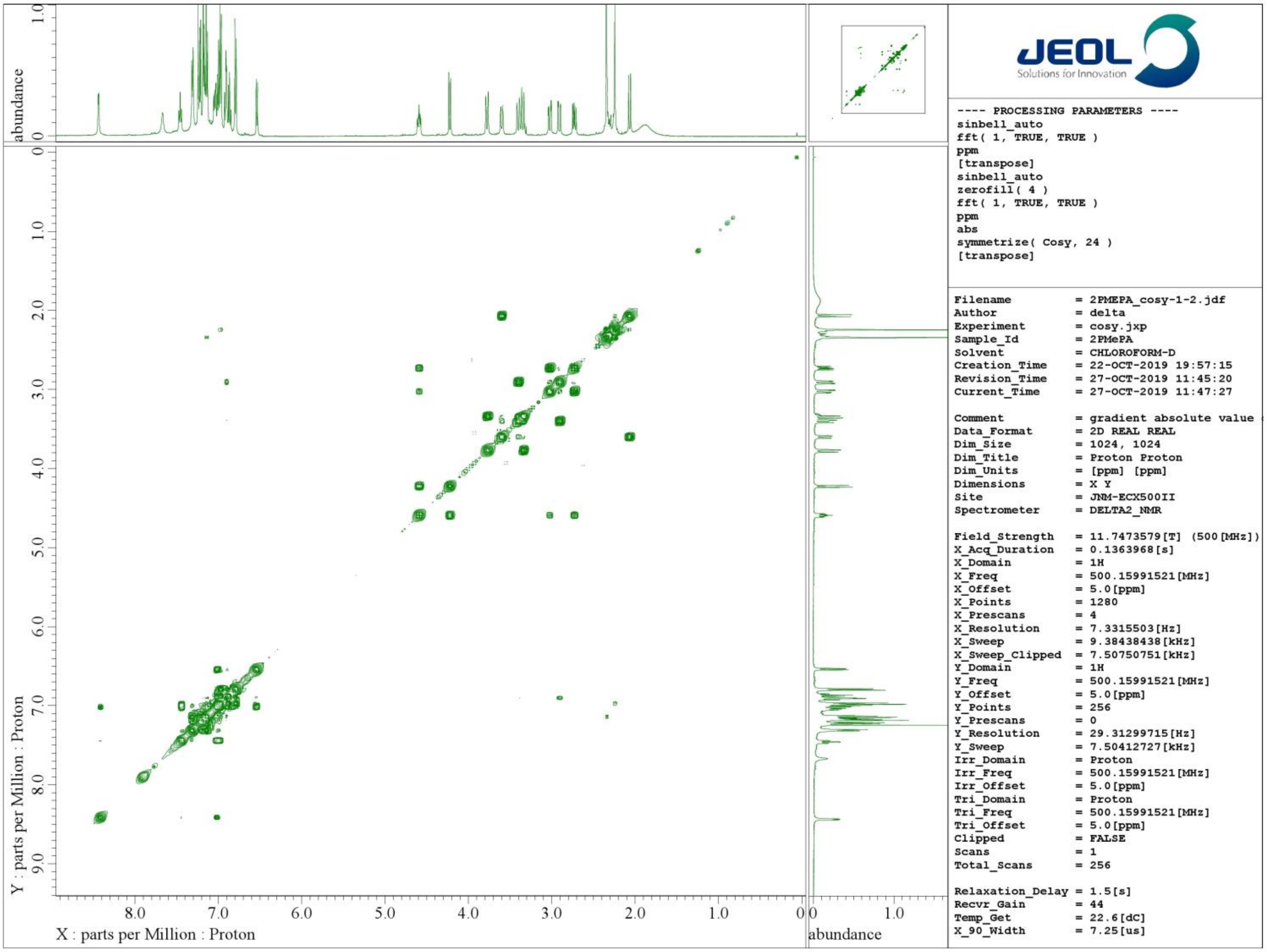

Fig. S4. COSY NMR spectrum of **8e**

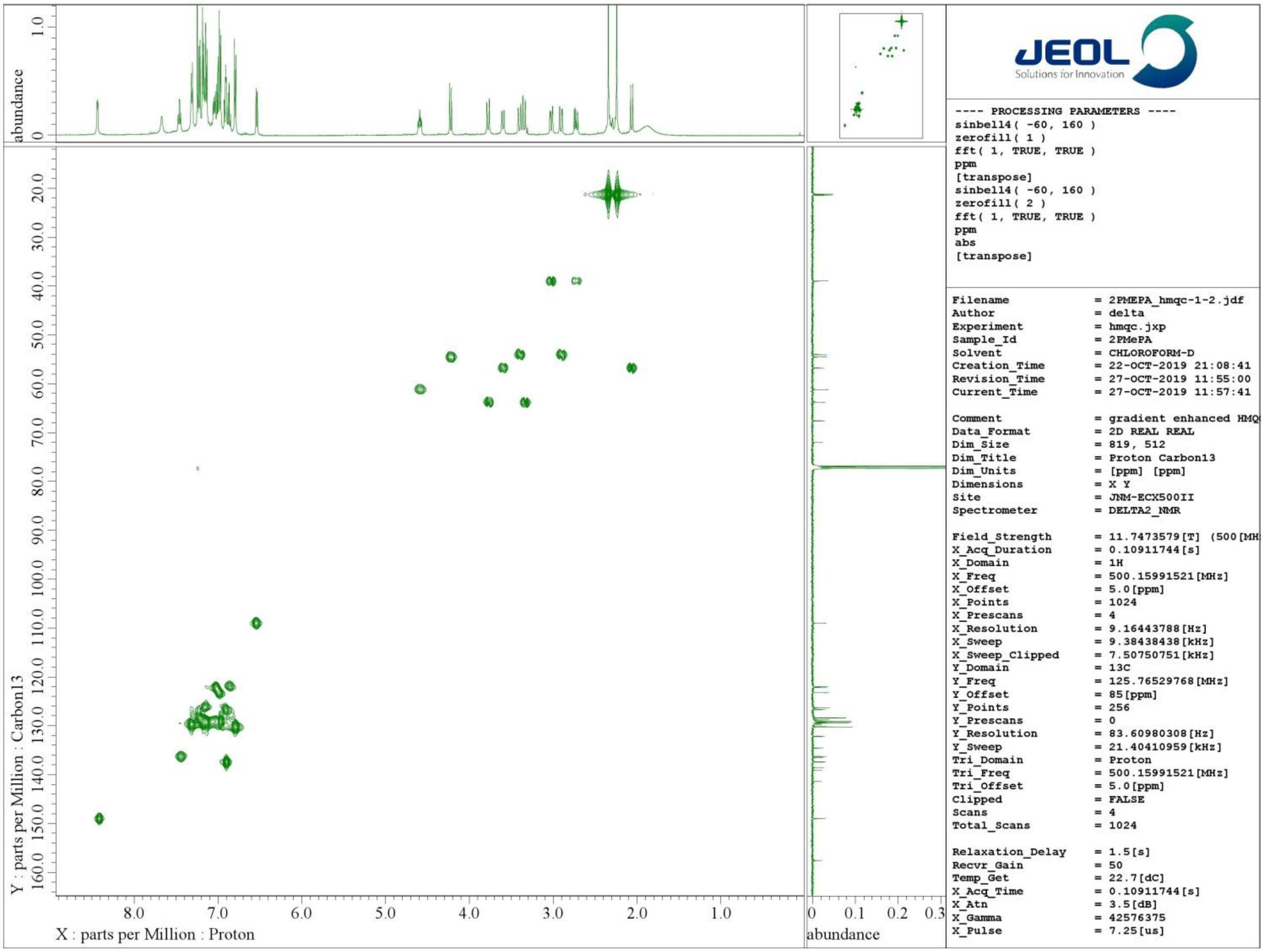

Fig. S5. HMQC NMR spectrum of **8e**

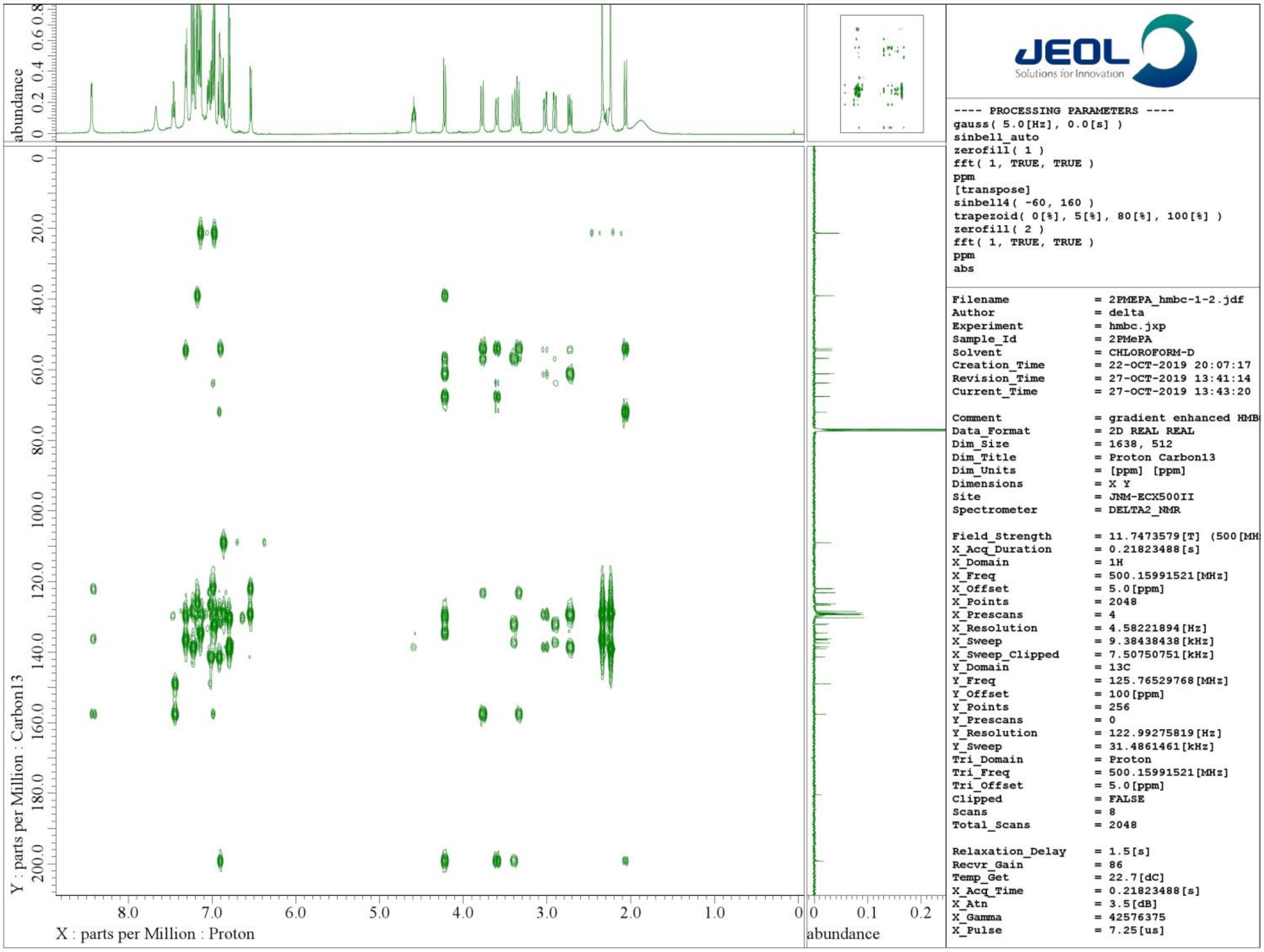

Fig. S6. HMBC NMR spectrum of **8e**

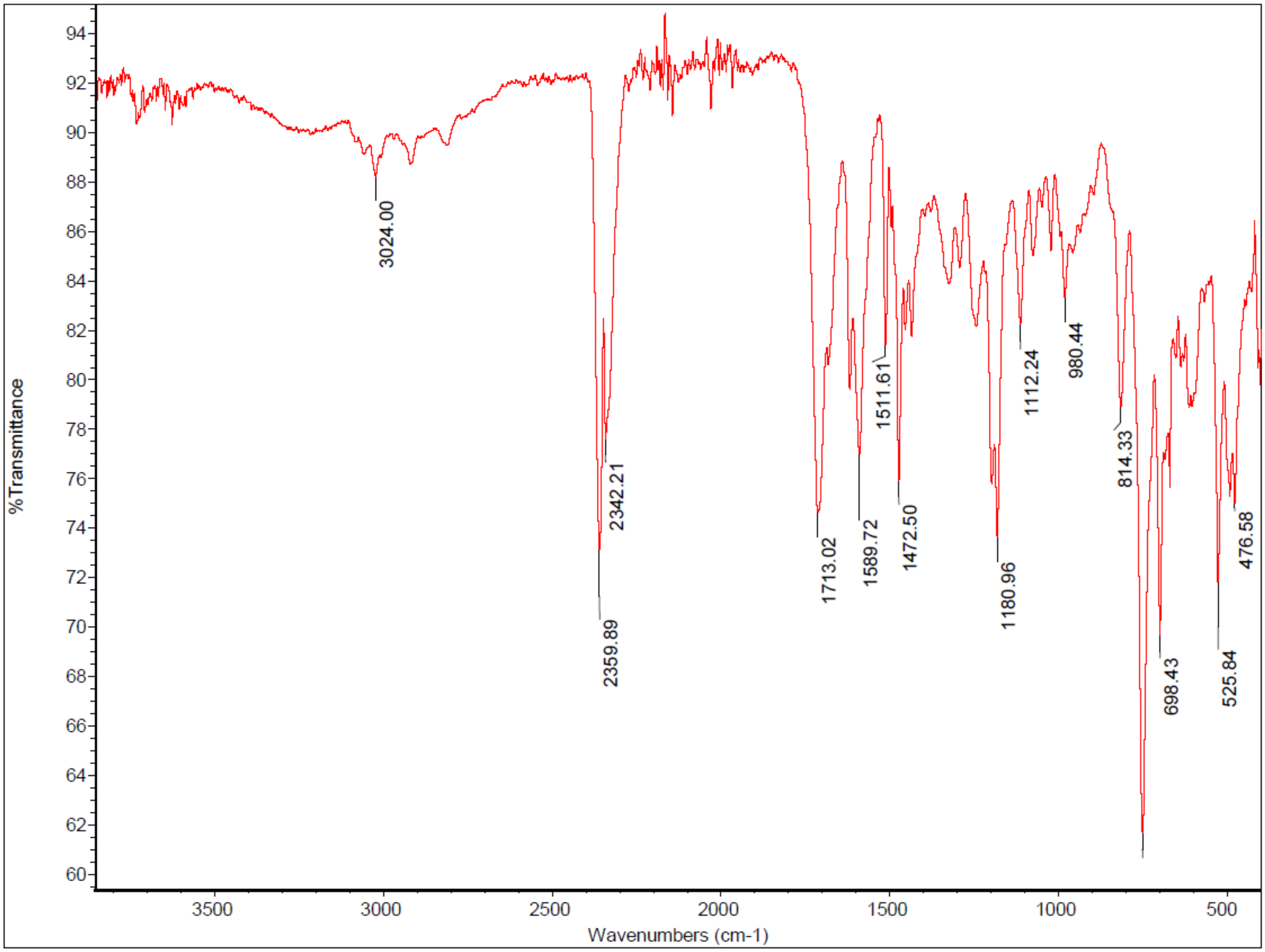

Fig. S7. FT-IR spectrum of **8e**
